# Dr. Partington

**Published:** 1854-05

**Authors:** 


### DR. PARTINGTON.

"Mrs. Partington" is an original creation; and the true one can be detected from her numerous imitators in a moment. The Rev. Sidney Smith first introduced this notable lady to the public, but *The Boston Post* is the only journal which records her original sayings and doings, which are only excelled—if, indeed, they are excelled at all—by Mrs. Lavinia Ramsbottom, the illustrious protegé of the witty Theodore Hook. Here are two of her late "utterances," which are quite as good, in their way, as anything in Madame Ramsbottom's letters from Rome or Paris:

"Diseases is very various—very. The Doctor tells me that poor old Mrs. Haze has got two buckles upon her lungs! It's dreadful to think of—'tis really. The diseases is so various! One day we hear of people's dying of 'hermitage of the lungs,' another of 'brown creatures;' here they tell us of the 'elementary canal' being out of order, and there about the 'tear of the throat;' hear we hear of the 'newrology in the head,' and there of an 'embargo' in the back. On one side of us we hear of a man getting killed by getting a piece of beef in his 'sarcofagus,' and there another kills himself by 'deskevering his jocular vein.' Things change so that I don't know how to subscribe for anything now-a-days. New names and 'rostrums' take the place of the old, and I might as well throw my old yerb bag away."

